# Procrastination and Health in Nurses: Investigating the Roles of Stress, Health Behaviours and Social Support

**DOI:** 10.3390/ijerph21070898

**Published:** 2024-07-10

**Authors:** Fuschia M. Sirois, Marios Biskas

**Affiliations:** 1 Department of Psychology, Durham University, Durham DH1 3LE, UK; 2 Department of Psychology, Bradford University, Bradford BD7 1DP, UK; m.biskas@bradford.ac.uk

**Keywords:** procrastination, nurses, stress, social support, health, health behaviours

## Abstract

**Objectives:** Evidence linking chronic procrastination to a range of poor health outcomes and trajectories continues to build. Yet, much of this research has been conducted in academic contexts or in non-student samples. Despite theory indicating that high-stress contexts increase vulnerability for procrastination, the pathways linking chronic procrastination to health outcomes proposed by the procrastination–health model have not been examined in a high stress environment. Accordingly, we tested the contribution of procrastination to health in nurses and whether social support was a protective factor. **Design:** Pre-registered cross-sectional study using a random sample of nurses recruited from the membership of a regional nursing association, supplemented by nurses and nurse trainees recruited from online nursing associations, conferences and forums. **Methods:** Nurses and nurse trainees (*N* = 597) completed measures of chronic procrastination, stress, health behaviours, social support and self-rated health. **Results:** Chronic procrastination was associated with perceived stress, health behaviours, self-rated health and social support in the expected directions. Consistent with the procrastination–health model, structural equation modelling revealed significant indirect effects linking chronic procrastination to poor self-rated health through higher stress and fewer health behaviours. Contrary to our hypotheses, social support did not moderate these pathways. **Conclusions:** This study is the first to demonstrate the relevance of procrastination for health in high-stress, non-academic contexts and to find support for both the stress and behavioural pathways linking procrastination to poor health outcomes. Findings further highlight the importance of addressing chronic procrastination as a vulnerability factor for poor health in nurses.

## 1. Introduction

Although procrastination is often considered in terms of its negative impact on productivity, research demonstrating the harmful effects of procrastination for health and well-being continues to grow. Defined as the voluntary and unnecessary delay of an intended task despite knowing that there will be negative effects for oneself and others for doing so [1,2], procrastination is a prevalent and pernicious issue that is linked to significant health issues, including poor health behaviours, higher stress, poor sleep, acute health problems [3,4,5,6,7,8,9] and even hypertension and cardiovascular disease [10]. According to the procrastination–health model [5,7], chronic procrastination confers risk for poor physical health-related outcomes due to the heightened stress and poor health behaviours associated with habitually procrastinating.

Apart from a handful of studies [7,10,11,12], much of this research has been conducted with student samples, due, in part, to the high prevalence of procrastination tendencies in this population [13]. However, new theoretical developments in understanding the factors that increase vulnerability for procrastination indicate that individuals in high-stress contexts are particularly prone to procrastination because such contexts deplete coping resources [14] and make it easier to use avoidant strategies such as procrastination for managing negative task-related mood. Social support is one well-known coping resource that can have beneficial effects for reducing stress and its harmful effects on health [15,16] and may therefore be useful for reducing procrastination in high-stress contexts. Despite this theory and research, there is little, if any, research investigating the potential role of social support for mitigating the effects of procrastination on health outcomes or in high-stress contexts. It is therefore unknown whether social support might buffer this stress and its harmful effects on health, or whether the effectiveness of social support is eclipsed by a high-stress context. The aim of the current study was to test the relevance of the procrastination–health model for understanding health outcomes in individuals working in a high-stress context, namely nursing, and whether social support might attenuate the negative impact of chronic procrastination on stress and health outcomes.

### 1.1. The Procrastination–Health Model

The routes through which chronic procrastination confers risk for poor health were first outlined in the procrastination–health model [5,7]. Grounded in theory and evidence linking personality to health outcomes [17,18], the procrastination–health model posited that elevated stress and poor practice of health-promoting behaviours are two key routes through which habitual procrastination increases risk for poor physical health. The stress-related pathway linking chronic procrastination to poor health is proposed to exert its damaging effects through its activation of stress-related psychophysiological pathways and the concurrent impact these have on immune system functioning, sleep, heart health, digestive system functioning, and other aspects of health related to elevated autonomic nervous system arousal [15]. The behavioural route, in contrast, is proposed to negatively impact health outcomes through avoidance and delay of health-promoting behaviours, such as healthy eating and regular exercise, and engagement in health risky behaviours. Although the impact of the behavioural route on health may not be immediately evident, the cumulative effects of poor health behaviours are well known to increase risk of disease and poor health especially amongst those with pre-existing vulnerabilities [19].

The evidence base for the procrastination–health model has continued to grow over the past two decades, with the proposed linkages of procrastination to health being demonstrated in several relevant populations. For example, chronic procrastination has been linked to higher stress, poor health behaviours and poor health-related outcomes in cross-sectional studies with undergraduate student samples [4,5,6,9,20,21]. Importantly, these links have been replicated in at least two longitudinal studies. In one population-based study, habitual procrastination was associated with higher stress, poor health behaviours and physical symptoms over time, although the proposed mediating pathways were not tested [8]. However, in a direct test of the procrastination–health model with 379 Canadian undergraduate students, stress but not health behaviours mediated the link between chronic procrastination and acute health problems across the academic term [3].

Although there has been less research testing the procrastination–health model in non-student samples, this evidence largely replicates the findings from that conducted with undergraduate students. For example, chronic procrastination was linked to lower physical activity across two cross-sectional studies conducted with community samples [11], and with higher stress and greater risk for having cardiovascular disease or hypertension in a large sample of community adults [10]. In a longitudinal study of community adults trying to make healthy changes including eating healthier and being more physically active, chronic procrastination was associated with less success in making intended health behaviour changes six months later [12]. Only one study to date has tested the proposed pathways of the procrastination–health model with a non-student sample. In this cross-sectional study, chronic procrastination was associated with lower self-rated health and both less frequent practice of health behaviours and higher perceived stress explained this link when tested separately [7]. However, when tested together, only stress explained significant variance in health.

### 1.2. The Complex Relationship between Procrastination and Stress

This theory and research on procrastination and health provides support for a causal relationship between chronic procrastination and stress. However, evidence also suggests this relationship is likely complex. For example, chronic procrastination is associated with stressogenic thoughts [21,22], and negative self-evaluations [23,24], both of which are known to contribute to stress. In this way these negative maintain and prolong the stress that occurs as a result of procrastinating [6,25].

Recent theoretical developments further implicate stress in the initiation and maintenance of procrastination through its contextual effects. Drawing on mood regulation perspectives of procrastination [2,26], the stress context vulnerability model of procrastination [14] posits that high-stress contexts provide insights into *when* people may be more likely to procrastinate. From this perspective, the difficulties in mood regulation that underscore procrastination will be particularly salient when an individual is dealing with contexts that create a backdrop of stress. These high-stress contexts increase vulnerability for procrastination in two ways. First, the coping demands of high stress contexts can deplete available coping resources (coping depletion hypothesis), making people more vulnerable to use procrastination as a means of emotion regulation for coping with difficult task-related emotions, as avoidant coping is a relatively low resource means of coping [15] 

Second, high stress contexts can lower the threshold for tolerating negative affective states (phenotypic vulnerability hypothesis) due in part to the cumulative effects of negative emotional experiences which can make minor difficulties feel more intense and difficult to handle. Additionally, stressful contexts contribute to lowered tolerance for negative states because stress compromises the very behaviours that can help bolster tolerance and resilience for stress, such as sleep [27]. The net effect is that individuals who are and who are not prone to procrastination under normal circumstances will be at greater risk for procrastination and its negative consequences for health and well-being in situations where background stress is high [14]. In turn, the stress that results from using procrastination as an avoidant coping strategy further adds to and amplifies the contextual sources of stress, creating a reciprocal and dynamic feedback loop that generates stress and maintains procrastination.

### 1.3. Social Support, Stress and Procrastination

Social support is one resource which may be particularly beneficial for reducing the harmful health effects of procrastination. Commonly defined as the different types of support that people receive from their relationships with others to help them deal with stressors [15], social support is a well-established resource for reducing stress in a variety of different contexts [28,29]. Despite the decades of evidence on the benefits of social support for reducing stress, there has been little if any research examining its effects with respect to chronic procrastination. However, theory and evidence indicate that people who chronically procrastinate experience higher levels of the negative social emotions such as guilt and shame, when procrastinating [30,31]. This suggests that despite the possible protective effects of seeking social support, individuals prone to procrastination may find it difficult to reach out for help, especially in high-stress contexts.

### 1.4. Procrastination, Stress, and Health in Nurses

One high-stress context which may be particularly relevant for examining the contribution of chronic procrastination to health is nursing. Nurses face a number of stressors in the context of their work including workload stress, stress from patients and their families, and stress from conflicts with supervisors and with peers [32], which can negatively impact their health [33,34]. When these stressors are combined with high workloads, low job control, poor social support, burnout, and poor quality of life can result [35], and compromise the quality of care they deliver [34]. Stress also has implications for the health practices of nurses. For example, a recent study found that 84% of nurses were chronically stressed, and 49% engaged in unhealthy behaviors, with higher levels of chronic stress contributing to less practice of health-promoting behaviours [36].

Given the high-stress context of nursing, not surprisingly emerging research has revealed that procrastination is a common issue among nurses that can further exacerbate their stress and compromise their health. For example, among Iranian nursing staff, 37% of nurses reported high and very high levels of procrastination [37]. Other research has found that chronic procrastination was associated with higher stress in a sample of nurses in Turkey [38], and with poor self-rated health and poorer health status in Iranian nurses [39,40]. With the exception of one qualitative study, there has been less research on procrastination and health behaviours in nurses. A study of nurses in Iran revealed that health-related procrastination, including delaying health protective behaviours, and avoiding seeking treatment for symptoms, was associated with prolonged recovery periods and experiencing acute and chronic health issues [39]. Although these findings are consistent with the procrastination–health model [5,7], a test of the complete procrastination–health model which exams both the health behaviours and stress pathways simultaneously in nurses is lacking.

### 1.5. The Current Study

Although a growing evidence base supports the proposed pathways linking chronic procrastination to poor health, to date there are no investigations of the procrastination–health model in high-stress, non-academic contexts. In addition, the potential protective role of social support on the known associations of chronic procrastination to stress, health behaviours, and health have yet to be tested. The aim of the current study was to address these important theoretical issues by testing the relevance of the procrastination–health model for understanding the health effects of procrastination in a high-stress context, namely nursing. Specifically, we expected that chronic procrastination would be negatively associated with engaging in health-promoting behaviours, and with self-rated health, and be positively associated with higher perceived stress. Additionally, we expected that stress and health behaviours would each account for the link between procrastination and self-rated health, as suggested by the procrastination–health model. Given that social support is a resource known to reduce stress in general and in nurses [41,42], we also examined the potential protective role of social support for mitigating the harmful effects of chronic procrastination on health. We expected that higher levels of perceived social support would weaken the proposed associations of chronic procrastination with stress and health behaviours, and health status.

## 2. Methods

### 2.1. Participants and Procedure

After receiving institutional ethical approval, 599 nurses and nurse trainees were recruited using two methods. First, a sample of 1000 nurses was randomly selected from a list of 6000 nurse members of the Ontario Nursing Association (Canada) and mailed a survey with a ‘thank you’ note and a tea bag inviting them to take time for themselves and complete the survey. A follow-up reminder was also mailed. From this sample, 264 nurses returned the survey (26.4% response rate). Second, a sample of 335 nurses and nurse trainees completed an online version of the same survey. The online sample was recruited by advertising the study on North American nursing organization web sites and message boards, and at a nursing conference. Data were collected pre-COVID19. Conformed consent was obtained from all participants.

From these participants, we excluded one participant who provided the same or no responses to most of the measures and one participant who was underaged. This yielded a final sample size of 597 participants (560 women, 37 men) aged between 18 and 72 years (*M* = 41.09, *SD* = 12.86). Participants were mainly white (92.8%), residents of Canada (71.8%) and nurses (88.3%) as opposed to nurse trainees.

### 2.2. Measures

In addition to demographic questions and questions about their professional status as a nurse or nurse trainee, participants completed the following measures.

**Social Support.** The Duke-UNC Functional Social Support [43] assessed perceived levels of personal social support. This scale consists of 8 items (e.g., “I have people who care what happens to me”) that are rated on a 5-point scale (1 = *As much as I would like* to 5 = *Much less than I would like*). Previous research has shown that the scale has good internal reliability (α = 0.86; [43]. After reverse scoring each item, we averaged responses to compute an overall score reflecting higher levels of perceived social support (α = 0.92, coefficient *H* = 0.94, *M* = 4.01, *SD* = 0.93).

**Procrastination**. Chronic or trait procrastination was assessed with the Revised Adult Inventory of Procrastination (AIP-R) [44]. This scale consists of 15 items (e.g., “I often think that I don’t get things done on time”) rated on a 7-point scale (1 = *strongly disagree* to 7 = *strongly agree*) and 5 distracter items not included in the overall scale score. Previous research has shown that the scale has good internal reliability (α = 0.84 [44]. After reverse scoring relevant items, item responses were averaged to compute an overall score reflecting higher levels of trait procrastination (α = 0.84, *H* = 0.88, *M* = 2.88, *SD* = 0.90).

**Stress**. We used three measures to assess different aspects of perceived stress to form the latent variable for the SEM analysis: (i) the Perceived Stress Scale (PSS) [45], which consists of 10 items assessing thoughts and feelings related to stressful events experienced (e.g., “In the last month, how often have you felt that things were going your way?”; 0 = *never* to 4 = *very often*), (ii) two items assessing perceived life stress experienced recently (i.e., “On average, how stressful has your life been in the past 2 weeks/6 months?”; 0 = *never* to 4 = *very often*) and (iii) two items assessing overall life stress (i.e., “I work or live with a lot of stress in my life,” “I feel I am under stress”; 1 = *strongly disagree* to 7 = *strongly agree*) which were included as distractor items in the AIP-R.

**Health Behaviours.** The Wellness Behaviours Inventory (WBI) [46] assessed the frequency that people engaged in common health-promoting behaviours (i.e., healthy eating, regular exercise, sleep behaviour and stress management). The WBI consists of 10 items (e.g., “I eat fresh fruits and/or vegetables”) rated on a 5-point scale (1 = *less than once a week or never* to 5 = *every day of the week*), along with 2 distractor items not included in the overall score for the scale. Previous research has shown that the scale has adequate internal reliability (α = 0.75) [7]. After reverse scoring relevant items, responses are averaged to compute an overall score reflecting more frequent practice of health-promoting behaviours (α = 0.74, *H* = 0.85, *M* = 3.57, *SD* = 0.66).

**Self-Rated Health.** We used three measures to assess different aspects of self-rated health to form the latent variable for the SEM analysis: (i) the single item global health rating question derived from the Medical Outcomes Survey 36 item short form health questionnaire [47] assessing general perceived health (i.e., “Please rate overall how your health is”; 1 = *excellent* to 5 = *poor*), (ii) two items assessing perceived health in comparison to other non-professional peers (i.e., “other people of your age,” “other people in your family”; 1 = *Very poor* to 6 = *Excellent*) and (iii) two items assessing perceived health in comparison to other medical staff (i.e., “other nurses,” “other doctors”; 1 = *Very poor* to 6 = *Excellent*).

## 3. Approach to Analysis

**Figure 1:**
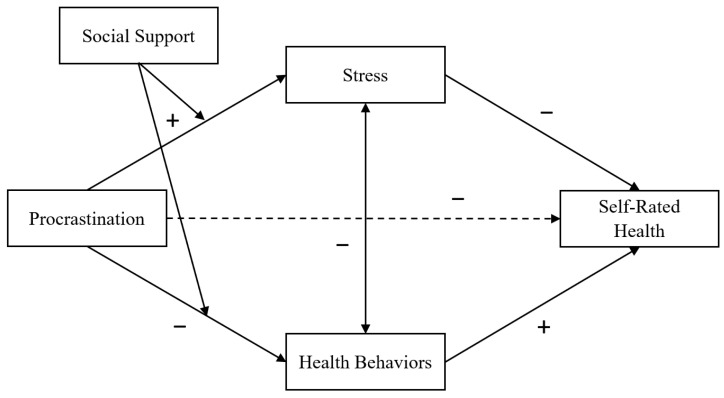
Proposed model of the procrastination–health model and the moderating role of social support. Dashed path indicates a proposed non-significant association after controlling for the contribution of the mediators.

We first computed correlations coefficients using SPSS 28.0 (IBM, Armonk, NY, USA) to examine the associations among the measured variables in the study. We then conducted structural equation modelling using AMOS 26.0 (IBM, Chicago, IL, USA) to test the hypothesized model (see Figure 1). We chose SEM for its ability to simultaneously test complex relationships among multiple variables, allowing for the examination of both direct and indirect effects within a single model while accounting for measurement error [45]. Additionally, SEM lowers the risk of Type I error which can be an issue when multiple separate analyses are run. As recommended by Kline [48], we first evaluated the measurement part of the model. Next, we added gender (coded 0 = male, 1 = female) and professional status (coded 0 = nurse trainee, 1 = nurse) as covariates to the model to investigate their associations with stress, health behaviours and self-rated health. Finally, we tested the full latent variable model. Specifically, we first tested a mediation model to examine the indirect effects of procrastination on self-rated health via stress and health behaviours. Next, we tested a moderated mediation model to examine whether social support moderated the aforementioned indirect effects.

The proposed model consisted of both multiple and single indicator factors. Specifically, we computed and specified the means of the PSS (α = 0.86, *H* = 0.91, *M* = 1.76, *SD* = 0.63), the measure assessing life stress experienced recently (*r* = 0.61, *M* = 6.06, *SD* = 2.06) and the measure assessing overall life stress (*r* = 0.68, *M* = 5.29, *SD* = 1.34) as the three indicators of stress. Similarly, we computed and specified the means of the measures assessing general perceived health (*M* = 3.60, *SD* = 0.86) and health in comparison to other non-professional peers (*r* = 0.78, *M* = 4.51, *SD* = 0.89) and medical staff (*r* = 0.88, *M* = 4.38, *SD* = 0.95) as the three indicators of self-rated health. Procrastination, health behaviours and social support were specified as single indicator factors. We used the means of the measures, instead of the individual items separately, as indicators of their respective factors to achieve a more parsimonious model and overcome problems that lengthy unidimensional scales often cause [48]. We also centred and multiplied the means of procrastination and social support to compute the interaction term between these variables. Finally, we correlated the residuals of stress and health behaviours in line with theory and previous research [7].

We estimated the parameters of the model using the Maximum Likelihood Method. We rejected the null hypothesis for the hypothesized main and interactive effects if *p* < 0.02 and for the (conditional) indirect effects if the 98% confidence interval (5000 bootstrap samples) did not include zero. We evaluated the fit of the models using two absolute fit indices: (i) the Chi-square test and (ii) the Root Mean Square Error of Approximation (RMSEA), and two incremental fit indices: (i) the Comparative Fit Index (CFI) and (ii) the Tucker–Lewis Index (TLI). Existing literature has indicated that the Chi-square test is sensitive to sample size [48]. As such, we mainly relied on RMSEA, CFI and TLI, which do not have a strong reliance on sample size, to test the fit of the models. We considered models with CFI and TLI values greater than 0.90, as well as RMSEA values below 0.10, to have acceptable fit [49,50]. 

## 4. Results

The study was registered before the analysis of the data on the Open Science Framework (OSF; https://osf.io/mxvtw). The dataset and all the models tested in the study are available on the OSF (https://osf.io/p92rx/?view_only=b006f2d61ed6484881fb0aaacb69a6cf).

### 4.1. Preliminary Analyses

**Missing data.** The percentage of missing data was 0.7%. We ran Little’s MCAR test to determine the distribution of the missing data. The test was significant, χ^2^ = 4233.56, df = 3943, *p* = 0.001, indicating that the data was not missing at random. As suggested by Enders [51], we used the multiple imputation technique to estimate the missing values.

**Correlations.** Trait procrastination was significantly associated with the three measures assessing stress, health behaviours and the three measures assessing self-rated health (see Table 1). Furthermore, the correlations among the measures assessing stress were significant, as were the correlations among the measures assessing self-rated health.

### 4.2. Main Analyses

**Measurement model.** The proposed model provided a good fit to the data, χ^2^(8) = 29.47, *p* < 0.001; CFI = 0.99, TLI = 0.98, RMSEA = 0.067. The factor loadings were statistically significant (*p* < 0.001) and above the recommended minimum of 0.50 [52], ranging from 0.75 to 0.93. Thus, we retained the proposed measurement model.

**Associations with gender and professional status.** Gender was not significantly associated with any factor. Regarding professional status, nurses exhibited lower stress (β = −0.12, *p* = 0.006) and higher self-rated health (β = 0.09, *p* = 0.022) compared to nurse trainees. We therefore included these significant associations in the subsequent models to adjust for the influence of professional status.

**Figure 2:**
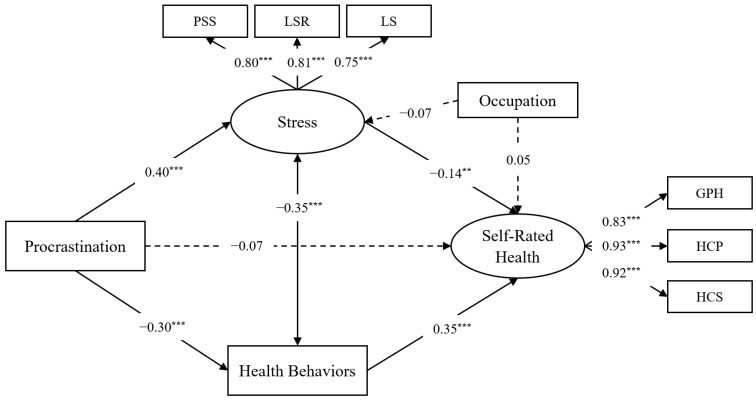
Mediation model of trait procrastination predicting self-rated health via perceived stress and health-promoting behaviours after controlling for professional role. Path coefficients are standardized. Dashed paths indicate non-significant associations. The latent variables are depicted in ellipses and the measured variables are depicted in rectangles. Error terms were estimated but omitted from the figure for clarity. *Note*. ** *p* < 0.01, *** *p* < 0.001. PSS = perceived stress scale, LSR = life stress experienced recently, LS = overall life stress, GPH = general perceived self-rated health, HCP = health in comparison to non-professional peers, HCS = health in comparison to medical staff.

**Mediation model.** The proposed model provided a good fit to the data, χ^2^(21) = 67.96, *p* < 0.001; CFI = 0.98, TLI = 0.96, RMSEA = 0.061. As illustrated in Figure 2, trait procrastination was significantly associated with greater stress and less frequent practice of health behaviours. In turn, stress was significantly associated with lower self-rated health, and health behaviours were significantly associated with higher self-rated health. The indirect effect of procrastination on self-rated health via stress was significant, *ab* = −0.043, *SE* = 0.020, 98% CI = [−0.093, −0.001].

Additionally, the indirect effect via health behaviours was significant, *ab* = −0.085, *SE* = 0.016, 98% CI = [−0.128, −0.053].

**Moderated mediation model.** The proposed model provided a good fit to the data, χ^2^(31) = 98.30, *p* < 0.001; CFI = 0.97, TLI = 0.95, RMSEA = 0.060. Perceived social support did not significantly moderate the link between procrastination and stress, β = 0.07, *p* = 0.068, or the link between procrastination and health behaviours, β = −0.01, *p* = 0.845. Furthermore, perceived social support did not significantly moderate the indirect effects of procrastination on self-rated health via stress, *B* = −0.008, *SE* = 0.005, 98% CI = [−0.026, 0.001], or health behaviours, *B* = −0.002, *SE* = 0.011, 98% CI = [−0.029, 0.025].

**Effect of sampling differences.** The conditional indirect effects remained non-significant when testing the model for participants who completed the survey online or on paper. In particular, multi-group analysis showed that the χ^2^ difference between the unconstrained (χ^2^ = 128.81, df = 62) and constrained (χ^2^ = 137.66, df = 66) models was non-significant, Δ χ^2^ (4) = 8.85, *p* = 0.065.

## 5. Discussion

The aim of the current study was to address two key limitations of previous research on the procrastination–health model [5,7] by testing the model in a high-stress context (i.e., nursing) and the potential role of social support for mitigating the harmful effects of chronic procrastination on health outcomes and pathways. Consistent with our hypotheses, the findings demonstrated that trait procrastination was related to higher perceived stress, less frequent practice of health-promoting behaviours and lower self-rated health. As expected, both higher stress and lower engagement in health-promoting behaviours each explained the association between trait procrastination and lower self-rated health. However, the findings did not support the proposition that social support moderated the pathways of the procrastination–health model.

The fact that we found that both the stress and health behaviour pathways explained the link between chronic procrastination and health is novel compared to previous research testing the procrastination–health model [5,7] and underscores the importance of considering the role that stressful contexts play in the contributions of chronic procrastination to health. Research with student and community adult samples has found that stress was the strongest pathway [3,5,7], with health behaviours only explaining significant variance when tested separately in non-student samples [7]. However, in the large sample of nurses tested in the current study, both pathways were significant. One possible explanation for this finding is that the behavioural pathway may be salient for understanding the ways in which chronic procrastination confers risk for poor health but only in high-stress contexts, non-academic contexts. Stress is known to be associated with poor health behaviours [53], such as an unhealthy diet [54,55] and less frequent exercise [54,56]. This is consistent with the small to moderate linkage between stress and health behaviours we found in the current study.

Alternatively, it may be that the importance of engaging in health-promoting behaviours for maintaining good health was particularly salient for nurses, suggesting that their perceptions of their self-rated health may have been impacted by this knowledge. Indeed, self-rated health theory indicates that a number of factors influence people’s perceptions of their health including awareness of their health practices [57]. Nonetheless, self-rated health is well known to be a robust predictor of morbidity and mortality, and correlates with other objective indicators of health [57]. So rather than being biased by their healthcare experience, it may be that nurses are more accurate in their assessment of their overall health status compared to student and community adult samples, and this contributed to finding a significant health behaviour pathway in the procrastination–health model [5].

Overall, the associations between chronic procrastination, stress, health behaviours and physical health status are consistent with the limited research that has been conducted on procrastination and health in nurses. This previous research, which was conducted with non-Western samples of nurses in Turkey and Iran, similarly found that chronic procrastination conferred risk for higher stress and poor health status, with some qualitative evidence of a link between chronic procrastination and poor health behaviours. The fact that we obtained similar results with a North American sample of nurses highlights the potential generalisability of our findings to non-Western contexts.

Contrary to what we hypothesized, social support was not a protective factor for the stress or behavioural pathways of the procrastination–health model. At the bivariate level, social support was associated with the model variables in the expected directions, most notably showing a moderate-sized negative association with stress. One possible explanation is the way in which social support was measured in the current study. The DUKE-UNC [43] focused on sources of personal social support rather than professional or organization social support. Previous cross-sectional research has noted the importance of general social support for addressing stress and burnout [35,42]. However, more rigorous longitudinal studies have suggested that organizational social support may be the more crucial form of social support for dealing with the effects of stress on nurses’ health [41]. Future research should therefore consider the context of that support to clarify whether or not it may be a protective factor with respect to procrastination and health.

### Limitations, Strengths and Future Directions

The findings of this study, though novel, should be considered with respect to several limitations and strengths. First, the cross-sectional nature of the study precludes drawing any firm conclusions about the temporal precedence of the relationships among the variables or about causality. Nonetheless, the proposed direction of the associations of chronic procrastination with stress and health behaviours, and, in turn, physical health status, are consistent with theory [5] and longitudinal research testing all possible directional pathways [3]. Second, the heterogeneity of the sample, which was comprised of both nurses and nursing students, limits the conclusions that can be drawn with respect to nurses in general, or for nursing students. Although over 88 percent of the sample were nurses, we did find that nursing students reported higher levels of stress and lower self-rated health compared to the nurses. This was likely due to the protective value of having more experience on stress, as more experience is associated with less stress in healthcare workers [58]. However, these differences were taken into account when testing the overall model and therefore their influence on the findings were minimal. Additionally, the procrastination–health model only accounts for the stress and behavioural pathways that can impact health, so it is unknown whether other explanations such as poor sleep might account for the health vulnerability linked to chronic procrastination.

One noteworthy strength of the current study was the random sampling of nurses from a professional nursing association. This robust approach supported a greater degree of representativeness of the sample than using only convenience sampling. Although part of the sample was garnered through notices placed on professional nursing sites, additional analyses revealed that the findings did not differ as a result of the sampling approach taken. Nonetheless, despite the robust sampling methods and incentives used, the response rate of 26.4% was somewhat low, although comparable to other studies conducted with nurses [59,60]. Time poverty is a clear barrier with respect to survey participation in nursing samples [61] and likely contributed to the low response rate in the current study.

Although our findings did not indicate that social support can help to reduce the risk for poor health associated with chronic procrastination in nurses, there may be other ways to address the health risks from chronic procrastination. According to the stress context vulnerability model of procrastination [14], the provision of coping resources may help address the coping depletion that can occur in high-stress contexts and increase vulnerability for procrastination. Such resources could focus on approaches such as mindfulness and other emotion regulation strategies that are known to reduce procrastination tendencies in other populations [62,63], as well as reduce stress and improve coping in samples of nurses [64,65,66].

## 6. Conclusions

Consistent with previous research on the procrastination–health model and theory underscoring the impact of high-stress contexts on the dynamic links between chronic procrastination and stress, we found that habitual procrastination was associated with poor self-rated health in nurses. Importantly, both higher stress and less frequent practice of health-promoting behaviours explained this linkage, which was not weakened by the presence of personal social support. From a theoretical perspective, these findings highlight the value of the procrastination–health model for understanding the health risks associated with chronic procrastination, even in populations who are aware of the importance of managing stress and health behaviours and who deal with ongoing background stressors. From a clinical perspective, the findings underscore the need to provide coping resources beyond social support to nurses as a means to reduce the risk of poor health outcomes from chronic procrastination.

Overall, these findings extend the procrastination–health model [5] by demonstrating the relevance of high-stress contexts for the behavioural route linking procrastination to for poor health and underscoring the need to address the health consequences of chronic self-regulation failure in contexts and populations with ongoing, high levels of stress. Given the high workloads and demands of nursing, the current findings underscore the need to recognize and address chronic procrastination as an important vulnerability factor for poor health in this population and to consider the provision of coping and emotion regulation training to tackle this issue sooner rather than later.

## Figures and Tables

**Table 1:** Correlations among the measured variables in the study.

		1	2	3	4	5	6	7	8	9
1	Trait Procrastination	--								
2	Social Support	−0.24 ***	--							
3	Perceived Stress Scale	0.39 ***	−0.40 ***	--						
4	Life Stress (last 2 weeks/month)	0.30 ***	−0.34 ***	0.64 ***	--					
5	Life Stress	0.26 ***	−0.36 ***	0.58 ***	0.63 ***	--				
6	Health Behaviors	−0.30 ***	0.33 ***	−0.35 ***	−0.31 ***	−0.36 ***	--			
7	General Self-rated Health	−0.23 ***	0.32 ***	−0.33 ***	−0.24 ***	−0.21 ***	0.41 ***	--		
8	Health Compared to Peers	−0.22 ***	0.26 ***	−0.27 ***	−0.22 ***	−0.15 ***	0.39 ***	0.77 ***	--	
9	Health Compared to Staff	−0.21 ***	0.32 ***	−0.28 ***	−0.24 ***	−0.17 ***	0.40 ***	0.76 ***	0.86 ***	--

*Note.* *** *p* < 0.001.

## Data Availability

The study was registered before the analysis of the data on the Open Science Framework (OSF; https://osf.io/mxvtw). The dataset and all the models tested in the study are available on the OSF (https://osf.io/p92rx/?view_only=b006f2d61ed6484881fb0aaacb69a6cf).

## References

[1] Ferrari J.R., Tice D.M. (2000). Procrastination as a self-handicap for men and women: A task-avoidance strategy in a laboratory setting. J. Res. Personal..

[2] Sirois F.M., Pychyl T.A. (2013). Procrastination and the priority of short-term mood regulation: Consequences for future self. Soc. Personal. Psychol. Compass.

[3] Sirois F.M., Stride C.B., Pychyl T.A. (2023). Procrastination and health: A longitudinal test of the roles of stress and health behaviours. Br. J. Health Psychol..

[4] Sirois F.M., Van Eerde W., Argiropoulou M.I. (2015). Is procrastination related to sleep quality? Testing an extension of the procrastination-health model. Cogent Psychol. OA.

[5] Sirois F.M., Melia-Gordon M.L., Pychyl T.A. (2003). “I’ll look after my health, later”: An investigation of procrastination and health. Personal. Individ. Differ..

[6] Sirois F.M. (2014). Procrastination and stress: Exploring the role of self-compassion. Self Identity.

[7] Sirois F.M. (2007). ‘‘I’ll look after my health, later’’: A replication and extension of the procrastination–health model with community-dwelling adults. Personal. Individ. Differ..

[8] Johansson F., Rozental A., Edlund K., Côté P., Sundberg T., Onell C., Rudman A., Skillgate E. (2023). Associations between Procrastination and Subsequent Health Outcomes Among University Students in Sweden. JAMA Netw. Open.

[9] Li X., Buxton O.M., Kim Y., Haneuse S., Kawachi I. (2020). Do procrastinators get worse sleep? Cross-sectional study of US adolescents and young adults. SSM Popul. Health.

[10] Sirois F.M. (2015). Is procrastination a vulnerability factor for hypertension and cardiovascular disease? Testing an extension of the procrastination-health model. J. Behav. Med..

[11] Kelly S.M., Walton H.R. (2021). “I’ll work out tomorrow”: The Procrastination in Exercise Scale. J. Health Psychol..

[12] Sirois F.M., Giguère B. (2018). Giving in when feeling less good: Procrastination, action control, and social temptations. Br. J. Soc. Psychol..

[13] Steel P. (2007). The nature of procrastination: A meta-analytic and theoretical review of quintessential self-regulatory failure. Psychol. Bull..

[14] Sirois F.M. (2023). Procrastination and Stress: A Conceptual Review of Why Context Matters. Int. J. Environ. Res. Public Health.

[15] Taylor S.E., Sirois F.M., Molnar D.M. (2020). Health Psychology.

[16] Szkody E., Stearns M., Stanhope L., McKinney C. (2021). Stress-Buffering Role of Social Support during COVID-19. Fam. Process.

[17] Suls J., Rittenhouse J.D., Friedman H.S. (1990). Models of linkages between personality and disease. Personality and Disease.

[18] Smith T.W. (2006). Personality as risk and resilience in physical health. Curr. Dir. Psychol. Sci..

[19] World Health Organization (2015). Global Status Report on Noncommunicable Diseases 2014.

[20] Sirois F.M., Tosti N. (2012). Lost in the moment? An investigation of procrastination, mindfulness, and well-being. J. Rat.-Emo. Cogn.-Behav. Ther..

[21] Flett G.L., Stainton M., Hewitt P., Sherry S., Lay C. (2012). Procrastination automatic thoughts as a personality construct: An analysis of the procrastinatory cognitions inventory. J. Rat.-Emo. Cogn.-Behav. Ther..

[22] Sirois F.M., Sirois F.M., Pychyl T. (2016). Procrastination, stress, and chronic health conditions: A temporal perspective. Procrastination, Health, and Well-Being.

[23] Flett G.L., Blankstein K.R., Martin T.R., Ferrari J.R., Johnson J.H., McCown W.G. (1995). Procrastination, negative self-evaluation, and stress in depression and anxiety: A review and preliminary model. Procrastination, and Task Avoidance: Theory, Research, and Treatment.

[24] McCown W.G., Blake I., Keiser R. (2012). Content analyses of the beliefs of academic procrastinators. J. Rat.-Emo. Cogn.-Behav. Ther..

[25] Smyth J., Zawadzki M., Gerin W. (2013). Stress and disease: A structural and functional Analysis. Soc. Personal. Psychol. Compass.

[26] Pychyl T.A., Sirois F.M., Sirois F.M., Pychyl T. (2016). Procrastination, emotion regulation, and well-being. Procrastination, Health, and Well-Being.

[27] Lund H.G., Reider B.D., Whiting A.B., Prichard J.R. (2010). Sleep patterns and predictors of disturbed sleep in a large population of college students. J. Adolesc. Health.

[28] Cohen S., Wills T.A. (1985). Stress, social support, and the buffering hypothesis. Psychol. Bull..

[29] Jolly P.M., Kong D.T., Kim K.Y. (2021). Social support at work: An integrative review. J. Organ. Behav..

[30] Fee R.L., Tangney J.P. (2000). Procrastination: A means of avoiding shame or guilt?. J. Soc. Behav. Personal..

[31] Giguère B., Sirois F.M., Vaswani M., Sirois F.M., Pychyl T.A. (2016). Delaying things and feeling bad about it? A norm-based approach to procrastination. Procrastination, Health, and Well-Being.

[32] Lee E.-K., Kim J.-S. (2020). Nursing stress factors affecting turnover intention among hospital nurses. Int. J. Nurs. Pract..

[33] Roberts R.K., Grubb P.L. (2014). The Consequences of Nursing Stress and Need for Integrated Solutions. Rehabil. Nurs..

[34] Sarafis P., Rousaki E., Tsounis A., Malliarou M., Lahana L., Bamidis P., Niakas D., Papastavrou E. (2016). The impact of occupational stress on nurses’ caring behaviors and their health related quality of life. BMC Nurs..

[35] Dall’Ora C., Ball J., Reinius M., Griffiths P. (2020). Burnout in nursing: A theoretical review. Hum. Resour. Health.

[36] Heuel L., Svea L., Ann-Kathrin O., Bettina W. (2022). Chronic stress, behavioral tendencies, and determinants of health behaviors in nurses: A mixed-methods approach. BMC Public Health.

[37] Babaie M., Farahani A.S., Nourian M., Hosseini M., Mohammadi A. (2022). Assessment of procrastination in providing nursing care among Iranian nursing staff. BMC Nurs..

[38] ŞAnlitÜRk D., Ardıç M. (2023). The Relationship Between Perceived Stress Levels of Nurses and Their General Procrastination: Cross-Sectional Study. Turk. Klin. J. Nurs. Sci..

[39] Basirimoghadam M., Rafii F., Ebadi A. (2020). Self-rated health and general procrastination in nurses: A cross-sectional study. Pan Afr. Med. J..

[40] Moghadam M.B., Basiri M., Ebadi A. (2019). Health-Related Procrastination in Nurses: Prevalence and Related Factors. Crescent J. Med. Biol. Sci..

[41] Bradley J.R., Cartwright S. (2002). Social Support, Job Stress, Health, and Job Satisfaction Among Nurses in the United Kingdom. Int. J. Stress Manag..

[42] Wu F., Ren Z., Wang Q., He M., Xiong W., Ma G., Fan X., Guo X., Liu H., Zhang X. (2021). The relationship between job stress and job burnout: The mediating effects of perceived social support and job satisfaction. Psychol. Health Med..

[43] Broadhead W.E., Gehlbach S.H., de Gruy F.V., Kaplan B.H. (1988). The Duke-UNC Functional Social Support Questionnaire: Measurement of Social Support in Family Medicine Patients. Med. Care.

[44] McCown W.G., Johnson J.L. (2001). The Adult Inventory of Procrastination Revised.

[45] Cohen S., Williamson G., Spacapan S., Oskamp S. (1988). Perceived stress in a probability sample of the United States. The Social Psychology of Health: Claremont Symposium on Applied Social Psychology.

[46] Sirois F.M. (2001). The Wellness Behaviors Inventory.

[47] Ware J.E.J., Sherbourne C.D. (1992). The MOS 36-item short-form health survey (SF-36): I. Conceptual framework and item selection. Med. Care.

[48] Kline R.B. (2016). Principles and Practice of Structural Equation Modeling.

[49] Hu L.-t., Bentler P.M. (1999). Cutoff criteria for fit indexes in covariance structure analysis: Conventional criteria versus new alternatives. Struct. Equ. Model..

[50] Browne M.W., Cudeck R. (1992). Alternative Ways of Assessing Model Fit. Sociol. Methods Res..

[51] Enders C.K. (2010). Applied Missing Data Analysis.

[52] Ford J., MacCallum R., Tait M. (1986). The application of factor analysis in psychology: A critical review and analysis. Pers. Psychol..

[53] Homan K.J., Sirois F.M. (2017). Self-compassion and physical health: Exploring the roles of perceived stress and health-promoting behaviors. Health Psychol. Open.

[54] Ng D.M., Jeffery R.W. (2003). Relationships between perceived stress and health behaviors in a sample of working adults. Health Psychol..

[55] Steptoe A., Lipsey Z., Wardle J. (1998). Stress, hassles and variations in alcohol consumption, food choice and physical exercise: A diary study. Br. J. Health Psychol..

[56] Stults-Kolehmainen M.A., Sinha R. (2014). The effects of stress on physical activity and exercise. Sports Med..

[57] Jylhä M. (2009). What is self-rated health and why does it predict mortality? Towards a unified conceptual model. Soc. Sci. Med..

[58] Sirois F.M., Owens J. (2021). Factors Associated With Psychological Distress in Health-Care Workers During an Infectious Disease Outbreak: A Rapid Systematic Review of the Evidence. Front. Psychiatry.

[59] Guise V., Chambers M., Välimäki M., Makkonen P. (2010). A mixed-mode approach to data collection: Combining web and paper questionnaires to examine nurses’ attitudes to mental illness. J. Adv. Nurs..

[60] Becker H., Cookston J., Kulberg V. (2000). Mailed survey follow-ups--are postcard reminders more cost-effective than second questionnaires?. West. J. Nurs. Res..

[61] VanGeest J., Johnson T.P. (2011). Surveying Nurses: Identifying Strategies to Improve Participation. Eval. Health Prof..

[62] Eckert M., Ebert D.D., Lehr D., Sieland B., Berking M. (2016). Overcome procrastination: Enhancing emotion regulation skills reduce procrastination. Learn. Individ. Differ..

[63] Rad H.S., Samadi S., Sirois F.M., Goodarzi H. (2023). Mindfulness intervention for academic procrastination: A randomized control trial. Learn. Individ. Differ..

[64] Ghawadra S.F., Abdullah K.L., Choo W.Y., Phang C.K. (2019). Mindfulness-based stress reduction for psychological distress among nurses: A systematic review. J. Clin. Nurs..

[65] Guillaumie L., Boiral O., Champagne J. (2017). A mixed-methods systematic review of the effects of mindfulness on nurses. J. Adv. Nurs..

[66] Smith S.A. (2014). Mindfulness-Based Stress Reduction: An Intervention to Enhance the Effectiveness of Nurses’ Coping With Work-Related Stress. Int. J. Nurs. Knowl..

